# Characterization of the Intra-tumoral B Cell Immunoglobulin Repertoire Is of Prognostic Value for Esophageal Squamous Cell Carcinoma

**DOI:** 10.3389/fimmu.2022.896627

**Published:** 2022-06-22

**Authors:** Zhizhong Wang, Zhenguo Cheng, Shuangshuang Lu, Louisa S. Chard Dunmall, Jun Wang, Yongjun Guo, Yaohe Wang

**Affiliations:** ^1^ National Center for International Research in Cell and Gene Therapy, Sino-British Research Centre for Molecular Oncology, State Key Laboratory of Esophageal Cancer Prevention Treatment, School of Basic Medical Sciences, Academy of Medical Sciences, Zhengzhou University, Zhengzhou, China; ^2^ Department of Molecular Pathology, The Affiliated Cancer Hospital of Zhengzhou University, Henan Cancer Hospital, Zhengzhou, China; ^3^ Department of Pharmacology, School of Basic Medical Sciences, Academy of Medical Sciences, Zhengzhou University, Zhengzhou, China; ^4^ Centre for Biomarkers Biotherapeutics, Barts Cancer Institute, Queen Mary University of London, London, United Kingdom; ^5^ Centre for Cancer Genomics and Computational Biology, Barts Cancer Institute, Queen Mary University of London, London, United Kingdom

**Keywords:** ESCC, TIBs, immunoglobulin repertoire, IgG2-producing plasma cells, prognosis

## Abstract

Esophageal Squamous Cell carcinomas (ESCC) is a highly heterogeneous malignancy that is among the leading cause of cancer-related death worldwide. B cells play pivotal roles in the immune defense system and cancer progression and regression, yet the repertoire of tumor infiltrating B cells (TIBs) and its association with clinical outcome remains unexplored in ESCC. Here we collected bulk RNA-seq sequencing data from 119 ESCC tumors and matched adjacent normal samples to delineate the B cell repertoire. We found that ESCC is more heavily infiltrated by B cells and plasma cells compared to activated T cells. The immunoglobulin heavy chain variable region (IGHV) gene usage was remarkably biased and *IGHV3-74* was under-represented in ESCC tumors. The TIBs showed a more oligoclonal profile along with widespread clonal expansion and IgG subclass switch events (CSRs). Survival analysis revealed several unexpected associations between tumor infiltrating B cells and prognosis. Higher levels of immunoglobulin expression (IGH), CD138 expression, IGH to *MS4A1* ratio, CSR events and clone diversity are all associated with better survival. Notably, we found that the abundance of *CD20*-negative IgG2-producing plasma cells has a strong positive effect on overall survival with a hazard ratio (HR) of 0.40 (log-rank p: 0.002). Combing molecular subtyping, the IgG2-producing plasma cells could stratify high-risk patients more accurately with a HR of 0.253 (log-rank p: 0.0006). The direct link between protective B cell populations and ESCC prognosis provides biomarkers for high-risk patient selection and holds great promise for developing strategies for immunotherapy targeting B cells in ESCC patients.

## Introduction

Esophageal Squamous Cell carcinomas (ESCC), which accounts for 80% of esophageal malignancies, is most prevalent in Eastern Asia and Eastern and Southern Africa (1). Although advances in diagnosis and treatment are being made, the five-year survival rate remains poor and it ranks sixth in mortality rates worldwide due to its invasive nature. Tumor suppressors, such as *TP53*, *FAT1*, *KMT2D* and *NOTCH1* are often aberrantly expressed in ESCC (2–4), yet limited progress has been achieved in the development of strategies to target these tumor suppressors and no effective molecular drugs are available for treatment of ESCC.

Immunotherapies using immune checkpoint inhibitors (5, 6), for example targeting the programmed cell death protein 1/programmed cell death ligand 1 (*PD-1/PD-L1*) axis, and cellular therapy including the use of chimeric antigen receptor (CAR) T cells (7, 8), have revolutionized the treatment of cancers (9, 10). The results from ESCORT-1^st^ clinical trials demonstrated that patients with unresectable, locally advanced, or metastatic ESCC who received camrelizumab (a PD-1 inhibitor) plus chemotherapy showed significantly prolonged overall survival (OS) and progression-free survival than those who received placebo plus chemotherapy. However, the combined treatment increased the rate of immune-related adverse events and a higher rate of severe adverse events, leading to treatment discontinuation in this study arm (11). Additionally, primary and secondary resistance to immunotherapy remains a barrier to long-term treatment success for the majority of cancer patients (12). Developing a full understanding of the complicated immune make-up of the tumor microenvironment (TME) in ESCC is crucial for developing novel, mechanistically-driven approaches to improve immunotherapy outcomes (13).

Single cell studies have revealed that in the ESCC TME, the dominant T cell population is exhausted, a finding that confounds the application of T cell based immunotherapy (14, 15). The role of tumor infiltrated B cells (TIBs) in anti-tumor immunity has, until recently, been underestimated. TIBs can account for up to 25% of all cells in some tumors, aggregate within tertiary lymphoid structures (TLS) in the TME and may trigger tumor cell clearance (16). TIBs might also serve as antigen presenting cells to promote anti-tumor helper T (Th) cell responses (16).

B cells have been described as having divergent roles in the TME, demonstrating both tumor-promoting and tumor-suppressing properties. The anti-tumor role of B cells in the TME can be attributed to the diversity of immunoglobulins (Igs) produced (17), which recognize foreign antigens and have shown high prognostic value in cancer (18). As antibodies directed towards intracellular tumor antigens are commonly observed in cancer patients (19), delineation of Ig repertoires in the TIBs can help us to understand how infiltrated B cells affect tumor development in multiple cancer types (20–24).

Whole transcriptome sequencing (RNA-seq) provides a powerful tool to dissect the interaction of cellular phenotypes and their molecular underpinnings (25). RNA-based biomolecules show promise for multi-faceted clinical applicability in various diseases, including cancers and infectious diseases (26–28). RNA-seq data provides a viable alternative to B cell receptor (BCR) targeted deep sequencing (BCR-seq) (20, 23, 29–31). Nonetheless, the BCR repertoire has not been systematically studied using RNA-seq data of ESCC. Recently, Zhang et al. reported distinct profiles of the TIBs in ESCC tumor samples compared with adjacent normal or peripheral blood samples from seven patients using multiplex PCR and high throughput sequencing (32), but no information regarding the predictive value in the diagnosis and prognosis of the disease was provided due to the small sample size analyzed.

We recently classified ESCC into four molecular subtypes that each displayed distinct gene expression profiles and TMEs (33). This offers opportunities for guiding therapeutic options. Here, we extend RNA-seq usage to assess the BCR repertoires of the tumor and matched adjacent normal samples in the largest ESCC series to date and provide an integrated analysis of TIBs and ESCC molecular subtypes. We reveal the previously unexpected prognostic significance of BCR lineages for stratification of ESCC. High levels of infiltrating *CD20*-negative IgG2-producing plasma cells are strongly associated with improved five-year survival rates. Our discoveries here improve our understanding of this disease at a genetic, molecular and cellular level to improve options for provision of individualized treatments and broadly for development of more effective therapeutic strategies for ESCC.

## Materials and Methods

### Patients and RNA-Seq Data Preparation

Clinical information and Sequencing materials of 119 patients diagnosed with esophageal squamous cell carcinoma (ESCC) were collected from Anyang Cancer hospital as described previously (33). Briefly, similar sized tumor and adjacent normal samples from each patient were used to extract RNA using Invitrogen’s TRIzol Regents according to the manufacturer’s instructions. After quantification with Agilent 2100 Bioanalyzer (Agilent RNA 6000 Nano Kit), 1 μg RNA was used to construct the sequencing library following the method provided by VAHTS^®^ Total RNA-seq (H/M/R) Library Prep Kit for Illumina^®^. Quantified libraries were sequenced using Illumina X Ten platform (BGI) with paired-end 150bp read length and at least 10 million paired-end 150bp reads for either tumor or matched adjacent normal tissue samples was generated. The raw sequencing data has been deposited at the National Genomics Data Center of China (https://bigd.big.ac.cn/) (Bioproject Access ID: PRJCA001577).

The gene expression values were calculated and represented as log2(FPKM +1). Briefly, raw sequencing data were first quantified against the indexed GRCh37 genome downloaded from Ensembl (34) using Salmon (version 0.9.0) (35). The quantified gene counts were loaded in an R 3.5 environment and genes without expression value in 50% of the samples were dropped. After that, the gene counts were normalized using the CQN (Conditional Quantile Normalization) pipeline (36) and the FPKM value was calculated using the normalized gene counts.

### B Cell Receptor Repertoire Detection

MixCR Software (20) was used to extract CDR3 repertoires from Fastq files. To minimize the technical error and maximize the confidence of comparative analysis, only samples with at least 500 reads mapping the Ig regions and CDR3s with clone counts ≥ 3 were used for further analysis. For V gene and J gene usage analysis, only one clone with an unambiguously mapped V or J gene was taken into account. The total clone abundance (N) was calculated as the sum of the clone counts of each clone. The clone fraction of each V gene was calculated using the clone counts of the V gene divided by N. To calculate rearrangements, we only considered the V J subgroups such as IGVH1, IGVH3, etc. The proportion of one V-J rearrangement was calculated as the sum of the clone fraction of each clone. When isotyping, clones mapped to *IGHG1*, *IGHG2*, *IGHG3*, and *IGHG4* were counted as IGHG, while clones mapped to *IGHA1* and *IGHA2* were recognized as IGHA. We also classified each subclass of IGHG and IGHA, counting those clones that only mapped to one of the subclasses.

To quantify the diversity of the BCR repertoires, clonality was calculated as 1 – normalized Shannon-Wiener index. A value close to 0 indicates an even distribution of clone fraction and more diverse BCRs. On the contrary, a value close to 1 indicates BCRs are more oligoclonal.

### Tumor-Infiltrating Immune Cell Deconvolution

According to the gene expression profiles of different lymphocytes, CIBERSORT (37) can deconvolve 22 immune cell subtypes. The detailed gene list used to define each immune cell type can be found at https://cibersort.stanford.edu. Firstly, we built a gene expression matrix as the CIBERSORT input mixture file. Each row of the matrix represents the expression (FPKM values) of a given gene and each data column consists of the expression profile for a single sample. Secondly, we updated the input mixture file to https://cibersort.stanford.edu/. Finally, the bioinformatic analysis was conducted online with the LM22 signature gene file (500 permutations).

### Survival Analysis

Survival analysis was undertaken using the survival (https://github.com/therneau/survival) and survminer (https://github.com/kassambara/survminer) packages in the R 3.5 environment. The cut-off value to split patients into two groups was determined using the surv_cutpoint function from the survminer package. All survival plots were generated using the Kaplan-Meier estimator. Plots were created with ggsurvplot function. Multivariable analysis was performed with Cox proportional hazard regression.

## Results

### Sequencing Profile of BCR and TCR With RNA-seq

**Figure 1:**
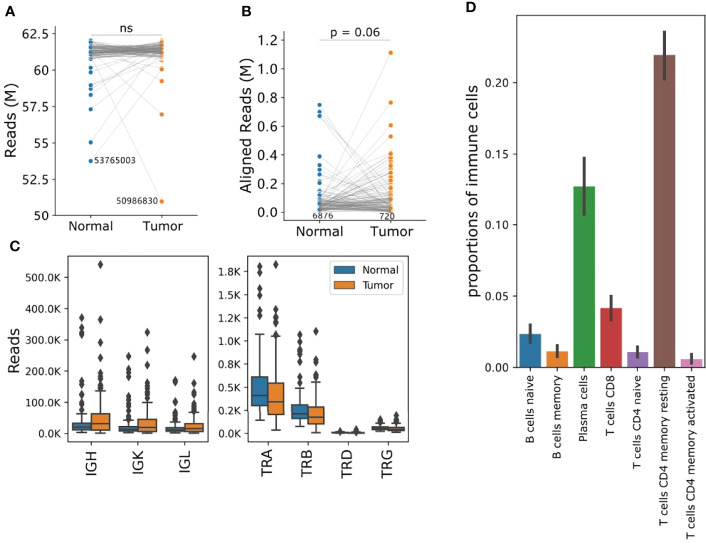
RNA-seq showed a unique TIB profile in the ESCC TME. **(A)** The total sequencing reads and number of paired-end reads per sample. **(B)** Reads can be mapped to the TCR or BCR regions. **(C)** Comparison of reads abundance of IGH, IGK, IGL, TRA, TRB, TRD and TRG. The y-axis unit is thousand. **(D)** Proportions of tumor-infiltrating B and T cells deconvoluted using CIBERSORT. Bars and error bars represent means and standard-errors, respectively. For a-b, the y-axis unit is million, p values were calculated using a Student t-test, and each blue dot is a normal sample, while red dots are tumor samples. The number in each plot refers to the minimum reads number for one sample in a group. IGH, Immunoglobulin Heavy Locus; IGK, Immunoglobulin Kappa Locus; IGL, Immunoglobulin Lambda Locus; TRA, T Cell Receptor Alpha Locus; TRB, T Cell Receptor Beta Locus; TRD, T Cell Receptor Delta Locus; TRG, T Cell Receptor Gamma Locus. ns, not significant.

As tumor infiltrating immune cells constitute only a small part of the tumor tissues, sequencing is key for discovery of TCR or BCR genes. In our dataset, at least 50 million paired-end reads were collected for each tumor or normal adjacent sample (**Figure 1A**). The number of reads successfully mapped to TCR or BCR regions ranged from 720 to 1.1 million without bias between tumor and related adjacent normal samples (**Figure 1B**). Notably, 97.84% ( ± 3.6) of aligned reads mapped to BCRs, and only a handful of reads mapped to TCRs (**Supplementary Table S1** and **Figure 1C**). Previous single cell RNA-seq investigating single cell TCR profiling (15) revealed that TCR clone type compositions and proliferative cell proportions were highly diverse across different T cell subtypes. For example,*CD4+* T cell subtypes had a low degree of clonal sharing and clone size. The diversity of TCR and BCR identified may reflect the differential immune cell infiltration in individual patient TMEs. In order to obtain global profiles of B cell and T cell-mediated immune responses in ESCC tumor, we further deconvolved the tumor-infiltrating immune cells using quantified gene expression data with CIBERSORT (37). ESCC was characterized by a predominant plasma cell and resting memory *CD4* T cell signature, but low infiltration of activated T cells (**Figure 1D**). Therefore, the low clone counts of TCRs in an immune suppressive ESCC tumor environment may be the cause of biased alignment of TCR and BCR reads. The insufficient T cell immunity and unique signature of B cell infiltrations in ESCC promoted us to further investigate the role of B cell immunity in disease progression and control.

### The Overall Characterization of V Gene and J Gene Usage

**Figure 2:**
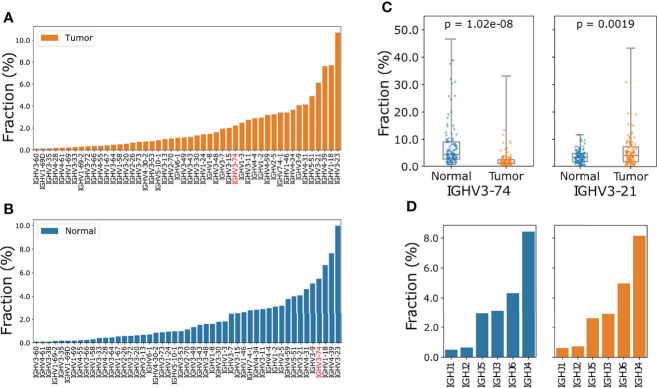
Comparison of IGHV gene and IGHJ gene usage in ESCC tumor and normal samples. **(A)** Ascending order of V gene frequency in ESCC tumor samples. **(B)** Ascending order of V gene frequency in matched normal samples. In **(A, B)** only V genes with frequencies large than 0.1% were included. The full spectrum of V gene usage is described in **Supplementary Table S2**. **(C)** Boxplot of *IGHV3-74* (left) and *IGHV3-21*(right) usage in normal and tumor samples, p values were calculated using Wilcoxon ranksum test. **(D)** Barplot of J gene usage of IGH chain for tumor (orange) and normal (blue) samples.

To minimize the effect of clones generated from technical error introduced by sequencing, we only took into account those CDR3 (third complementarity determining region of the heavy chain) clone records with no ambiguous mapping and at least 3 supporting reads for V J usage analysis. A total of 71 or 70 IGHV genes were identified in tumor and normal samples, respectively. The IGHV gene usage is very biased as the top five genes account for about 37.14% in tumor samples: *IGHV3-23* (10.71%), *IGHV1-18* (7.73%), *IGHV4-39* (7.64%), *IGHV3-21* (6.14%) and *IGHV5-51* (4.92%) (**Figure 2A**). When compared to the normal samples, we observed a higher proportion of *IGHV3-21* (median usage: 6.14% vs 4.09% (tumor vs normal tissue) student t-test p: 0.00019) and lower proportion of *IGHV3-74* usage in tumor samples (median usage: 2.23% vs 5.49%, (tumor vs normal tissue) student t-test p: 1.02e-8) (**Figures 2B, C**). *IGHJ4* and *IGHJ6* were the most used J segments, and the usage of 6 J segment genes was consistent between tumor and normal tissues (**Figure 2D**). Finally, we investigated the usage of V genes in IGK and IGL chains. Although similar V gene usage distribution between tumor and normal tissue were observed, the usage of IGK and IGL V genes was also greatly biased. There were 31 IGK V genes and 46 IGL V genes in the ESCC tumor samples and the usage proportion of the top 5 V genes was 59.76% and 48.48%, respectively (**Supplementary Figure S1**).

### V(D)J Recombination and CDR3 Properties

**Figure 3:**
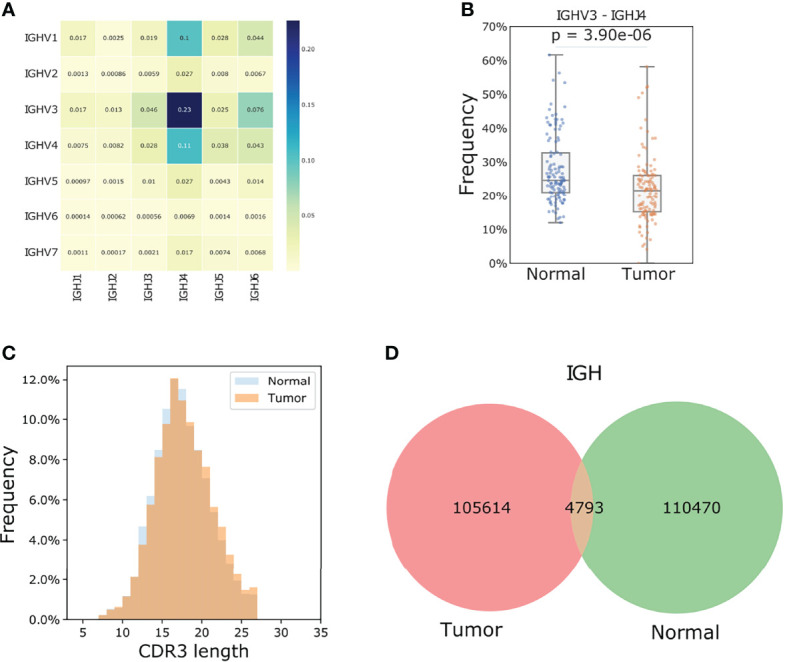
TIBs in ESCC are different populations compared to those found in matched normal samples. **(A)** V J rearrangement of tumor samples for the IGH chain, the number of each cell represents the corresponding fraction of each recombination. **(B)** IGHV3/IGHJ4 is the most frequent rearrangement and the mean frequency in each paired tumor and normal samples showed a significant difference. The p value was calculated using a Students t-test. **(C)** IGH chain CDR3 length distribution for the tumor samples. **(D)** Shared IGH CDR3s between tumor and adjacent normal samples.

Because of the limited number of sequencing reads mapped to the BCRs compared to the diversity of the rearrangement, we only studied V gene subgroups to increase the confidence of analysis. *IGHV3* (tumor: 40.3%, normal: 44.4%) and *IGHV4* (tumor: 23.1%, normal: 23.6%) were the most frequently used heavy chain V genes (**Figure 3A**). *IGHV3*/*IGHJ4* rearrangements are most common in ESCC tumors yet showed significantly lower usage compared to normal samples with a median frequency of 21.5% vs 24.6% (tumor vs normal tissue; student t-test p: 3.76e-06, **Figure 3B**).

In the 119 tumor samples, 105614 unique IGH CDR3 amino acids were found. Neither the CDR3 length distribution nor the clone abundance was different between tumor and normal samples, yet wide spectrums of CDR3 clone counts were observed among individuals (**Supplementary Figure S2**, **Figures 3C, D**). Only 4.5% of the IGH CDR3 clones were shared between tumor and normal samples (**Figure 3D**), which is much lower than that of IGL (14.5%) and IGK (35.8%) chains. We further calculated Jaccard Index for each paired tumor and normal sample. This index ranges from 0 to 1 and the higher the percentage, the more similar the two populations. Results showed that the B cells in tumors and the matched normal samples were almost two distinct populations with extremely low similarities as the median IGH Jaccard index was 0.006. In line with the amount of shared CDR3 clones, the Jaccard index for IGK and IGL chains was significantly higher than the heavy chain (Student t-test p< 0.0001, **Supplementary Figure S2**).

### ESCC TIBs Tend to be Oligoclonal and IgG Predominates

**Figure 4:**
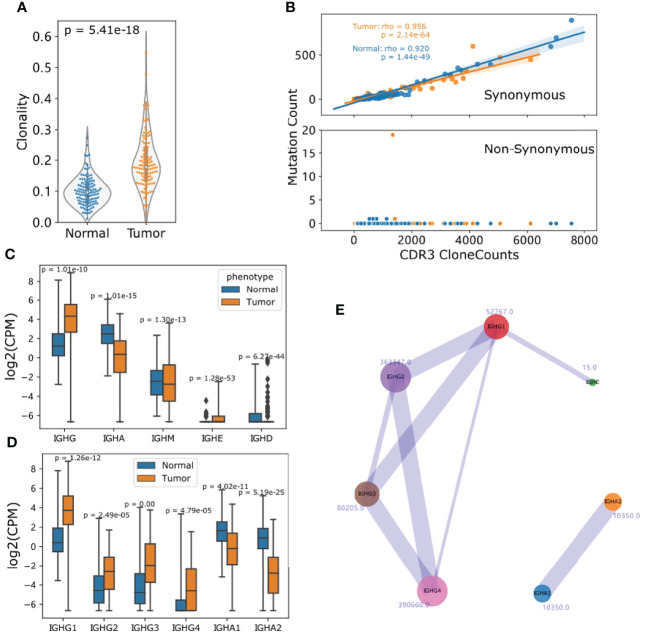
TIBs in ESCC predominately produce IgGs and are more oligoclonal than those in normal samples. **(A)** IGH clonality was calculated as 1–normalized Shannon-Wiener index with IGH CDR3 clones. **(B)** SHM profile of CDR3 clones, the x axis represents the CDR3 clone counts for each tumor and normal sample, y axis represents the mutated clones. **(C)** The CPM distribution of detected Ig isotypes for normal and tumor samples. **(D)** CPM distribution of subclasses of IGHG and IGHA. **(E)** Visualization of Ig isotype co-occurrence in the two corresponding Ig subclasses. The number beside the circle is the clone number and line widths are relative to the occurrence. For **(C, D)**, p Values were calculated using Student t test.

We calculated the 1 - normalized Shannon-wiener index of all IGH Chain CDR3s considering both CDR3 contents and frequency as a representation of clonality. Intriguingly, tumor samples showed a more oligoclonal profile of B cells compared to matched adjacent normal samples (Student t-test p: 5.41e-18) as the high-frequency clonotypes were exclusively observed in tumor samples (**Figure 4A**). When one base mismatch or deletion was defined as the main type of CDR3 somatic hypermutation (SHM), the median mutation ratio for tumor samples is 4.7%, significantly higher than that of normal samples (Wilcoxon ranksum p: 0.01). There was no association of SHM status between tumors and the corresponding normal samples (**Supplementary Figure S3**). In agreement with findings in normal samples and a previous report (23), the mutational load in tumor samples was positively associated with clone abundance (Spearman rho: 0.956) and the majority of mutations were synonymous (**Figure 4B**).

We defined CDR3 clones mapped to *IGHG1*, *IGHG2*, *IGHG3* and *IGHG4* as IGHG repertoires, *IGHA1* and *IGHA2* as IGHA repertories. To minimize the measurement deviation caused by sequencing depth, clonotypes per one million sequencing reads (CPM) (38) was introduced to quantify each Ig subclass. CPMs were highly consistent with RNA-seq-quantified FKPMs for all Ig subclasses (**Supplementary Figure S4**). Compared with normal samples, TIBs in ESCC showed a clear predominance of IGHG in most cases. Nonetheless, the CPM distribution for each IgG subclass showed differences and IgG1 quantification was much higher than other IgGs (**Figures 4C, D**). Aside from the constraints of RNA-seq data and expression differences, this may also be caused by subsequent class switch recombination (CSR) (23). When only clones mapped to two different subclasses were taken into account, we found that 18322 *IGHG4* clones co-existed with other subclasses, and IgG2/4 (7.76%), IgG3/1 (3.14%) ranked as the top two combinations (**Figure 4E**).

### Immunoglobin Characteristics Are Associated With ESCC Clinical Outcomes

**Figure 5:**
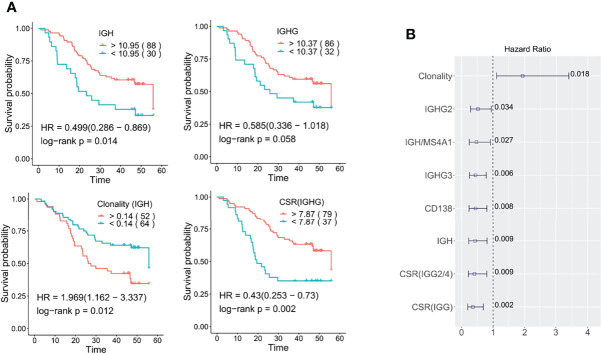
Role of tumor-infiltrating Ig profiles in ESCC prognosis. **(A)** Kaplan-Meier overall survival plots relating survival with IGH, IGHG, clonality, and IgG CSR. The numbers in brackets correspond to the patient number in each group **(B)** The prognostic significance of B cell characteristics was determined by the multivariable Cox proportional-hazards model. The numbers marked are the log-rank p values. Here IGHG is a sum of the expression of *IGHG1*, *IGHG2*, *IGHG3* and *IGHG4*; IGH is the sum of *IGHA1*, *IGHA2*, *IGHG*, *IGHE*, and *IGHD* genes. The cut off for all Kaplan-Meier curve were defined by the surv_cutoff function from the survminer package in the R3.5 environment, the min proportion was set to 0.15.

We first examined the possible prognostic value of the expression level of B cell antibodies. Patients were split into low and high expression groups according to the gene expression distribution, and at least 20 patients were included in each group. Kaplan-Meier analysis showed that higher expression of IGH (a sum of the FPKM values of *IGHG1*, *IGHG2*, *IGHG3*, *IGHAG4*, *IGHA1*, *IGHA2*, *IGHD*, and *IGHM* genes) is significantly associated with longer overall survival with a hazard ratio (HR) of 0.499 (log-rank p: 0.014, **Figure 5A**). The expression level of IGHG2, IGHG3, and IGHG (a sum of the *IGHG1*, *IGHG2*, *IGHG3*, *IGHAG4*) also had a positive effect on survival (**Supplementary Figure S5** and **Figure 5B**).

Secondly, we examined the prognostic potential of B cell clonality and class-switch recombination (CSR). Kaplan-Meier survival curve analysis showed that higher clonality (lower diversity) was negatively associated with prolonged survival with a HR of 1.97 (log-rank p: 0.012, **Figure 5A**). As to CSR, we also confirmed that high levels of CSR are significantly associated with longer survival (log-rank p: 0.002, **Figure 5A**). Regardless of the important role of IgG1/IgG3 isotypes in initiating tumor-specific antibody-dependent cellular cytotoxicity (ADCC), high IGHG3/1 class switch events were a neutral survival parameter in ESCC. On the other hand, IGHG2/4 and IGHG1/2 class switch events are significantly associated with better prognosis (log-rank p: 0.004, 0.034) (**Supplementary Figure S6**). A multivariate analysis using Cox proportional-hazard regression with adjustment for gender, age, smoking, drinking, tumor stage and infiltration was further conducted and confirmed the prognostic association of the above B cell characteristics (**Figure 5B**).

### IgG2-Producing Plasma B Cells in the TME Are Positively Associated With Longer Survival in ESCC

**Figure 6:**
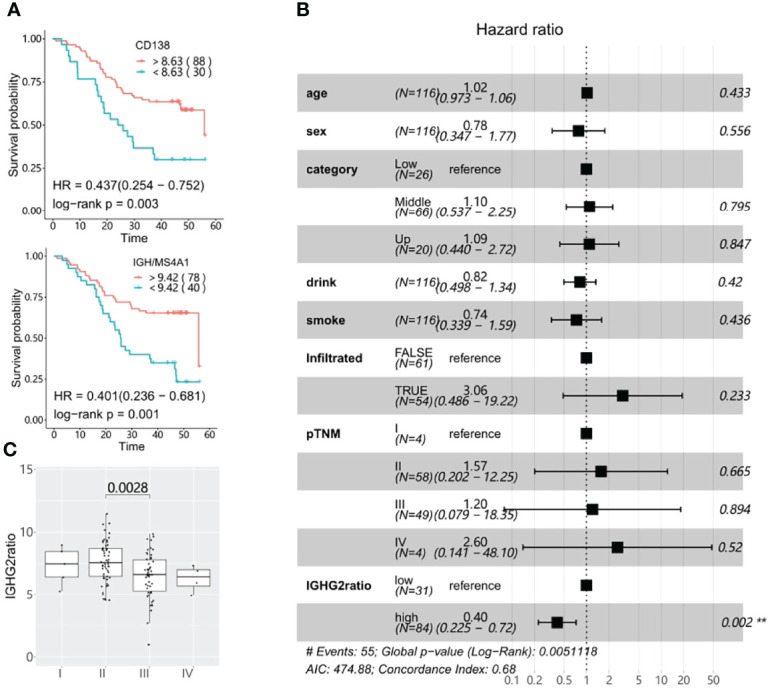
Survival significance of tumor-infiltrating plasma cells. **(A)** *CD138* expression and high *IGH* (the sum of *IGHA1*, *IGHA2*, *IGHG*, *IGHE*, and *IGHD* genes) to *MS4A1* ratios are associated with better prognosis in ESCC as determined using Kaplan-Meier overall survival plots. The numbers in brackets correspond to the patient number in each group. **(B)** The prognostic association of the *IGHG2* to *MS4A1* ratio(*IGHG2*ratio) was determined using the multivariable Cox proportional-hazards model. **(C)** The IGHG2 ratio distribution in different pTNM stages. The cut off for all Kaplan-Meier curve were defined by the surv_cutoff function from the survminer package in the R3.5 environment, the min proportion was set to 0.15.

The main TIBs resolved by CIBERSORT were plasma cells. We further assessed the prognostic value of the plasma cell marker *CD138* and non-plasma B cell marker *MS4A1* (encoding *CD20*). In line with another study (39), high expression of *CD138* was strongly beneficial for ESCC patient survival (log-rank p: 0.003). This association became more significant when we examined the ratio of IGH to *MS4A1*, which reflects the relative abundance of antibody-producing plasma cells compared to *CD20*-positive non-plasma B cells (log-rank p: 0.001, **Figure 6A** and **Figure 5B**). Since the Ig isotypes showed varied prognostic values in ESCC, we further assessed the ratio of isotype-specific plasma cells to non-plasma cells with multivariable Cox proportional-hazards regression and found that IgG1/3/4 expressing plasma cells had a neutral effect on prognosis, but high levels of IgG2-expressing plasma cells, determined by the ratio of *IGHG2* to *MS4A1*(IGHG2 ratio), was strongly associated with better survival (HR: 0.40, **Figure 6B**). We further explored the association between IGHG2 ratio and pathological characteristics and found that the IGHG2 ratio significantly decreased in III/IV stage patients compared with stage I or II patients (**Figure 6C**). High expression of *IGHG2* is also a positive survival parameter in different solid tumors when validated using TCGA (The Cancer Genome Atlas) data, including head and neck squamous cell carcinomas (TCGA-HNSCC) and breast invasive carcinoma (TCGA-BRCA) (**Supplementary Figure S7**). Taken together, this data suggests that IgG2-expressing plasma cells play an important role in B cell anti-tumor immunity in ESCC.

### Differences in TIB Characteristics Within ESCC Molecular Subtypes Can Better Indicate Prognosis

According to differences in gene expression profiles, we recently classified ESCC into four molecular subtypes: differentiated, characterized by keratinocyte differentiation and epidermis development; immunogenic, characterized by B-cell surface markers and T-cell chemokine ligands; metabolic, defined by upregulation of genes involved in drug metabolism by cytochrome P450 and retinol metabolism; and stemness, associated with stem cell markers and the *Wnt* signaling modulator *SFRP1*. Each molecular subtype was found to contain a very different TMEs and subtypes are strongly associated with prognosis (33).

**Figure 7:**
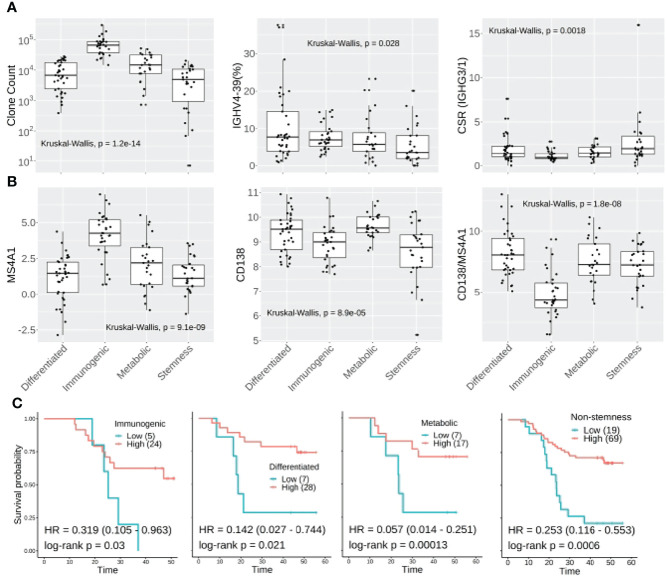
Molecular subtyping combined with TIB analysis could improve prognostic prediction in ESCC. **(A)** CDR3 clone abundance, *IGHV3-74* and IgG3/1 CSR showed different distributions amongst the four molecular subtypes of ESCC. **(B)** The expression distribution of *MS4A1*, *CD138* and *CD138*:*MS4A1* ratio among the four molecular subtypes. **(C)** Kaplan-Meier survival plot for non-stemness molecular subtypes. For the Kaplan-Meier plot, the patients were classified into IGHG2ratio high or low groups using surv_cutoff function from the survminer package in the R3.5 environment, then integrated with the molecular subtyping.

In line with gene expression profiles of the molecular subtypes (33), TIBs were most abundant in the immunogenic subtype (median CDR3 clones: 65351), whereas the stemness subtype (median: 4888) was much less B cell infiltrated (Kruskal–Wallis test p: 1.2e-14). We also observed the *IGHV4-39* usage proportion (median: 3.63%) was significantly decreased in stemness samples (Kruskal–Wallis test p: 0.028). No statistical differences were found for IGHV3-74 frequency, clonality, IgG2, or IgA CSR ratio among the four molecular subtypes, yet the stemness samples had a higher IgG3/1 ratio compared to other subtypes (Kruskal–Wallis test p: 0.0018) (**Figure 7A**). In addition, the immunogenic samples had the highest expression of *MS4A1* but lowest *CD138* to *MS4A1* ratio, suggesting that the TIBs in the immunogenic subtype are mainly *CD20* positive non-plasma cells. Although the differentiated and metabolic subtypes of ESCC have higher *CD138* expression than immunogenic and stemness subtypes, the *CD138* to *MS4A1* ratio of stemness was comparable with differentiated and metabolic subtypes (**Figure 7B**).

When we investigated the *IGHG2* to *MS4A1* ratio between subtypes, no significant difference was observed. However, multivariable Cox analysis confirmed that IgG2 level could further stratify the high-risk patients *within* immunogenic (HR: 0.319, log-rank p: 0.03), differentiated (HR: 0.142, log-rank p:0.021) and metabolic subtypes (HR: 0.057, log-rank p: 0.00013) (**Figure 7C**). As to the stemness subtype, which has the worst clinical outcome, the IgG2 plasma cell level showed no association with better survival (log-rank p: 0.3). Another multivariable Cox analysis was further conducted for non-stemness patients, resulting in a hazard ratio of 0.253 (log-rank p: 0.0006, **Figure 7C**).

Thus, combining the molecular subtyping and IgG2 expressing plasma cells in the TME could greatly benefit ESCC patient screening by determining their relative risk.

## Discussion

Although target-specific sequencing experiments can provide more information about the genomic features of disease, development and prognosis, a sufficient amount and quality of tumor samples are not always available for molecular analysis. Furthermore, laborious operations and high costs are obstacles to this approach (25). Whole exome or targeted DNA sequencing (40) and RNA-seq are the most widely used technologies in tumor research and can be used to derive a deeper understanding of the genetic and molecular subtypes of tumors that can play an important role in therapy design and subsequent clinical translation.

Taking advantage of bioinformatics software development and sequencing reagent optimization, the application of RNA-seq has extend to BCR analysis with high fidelity (20, 23, 29, 31). High levels of tumor-infiltrating B cells exist in many cancer types and have shown both positive and negative associations with clinical outcomes (17, 24, 32, 41, 42). Though the TME of ESCC has been analyzed in several single cell sequencing studies (14, 15), BCR characterization in the ESCC TME and its clinical association has not been investigated in large numbers of patient samples. In this study, we analyzed the BCR repertoire of 119 ESCC tumor samples and matched adjacent normal samples using bulk RNA-Seq data, and found an association of BCRs and antibody subtypes with prognosis. To the best of our knowledge, this work is the first to analyze the infiltrating B cell repertoires and their association with prognosis in a large cohort of ESCC patients.

Inter- and intra-tumoral heterogenicity make ESCC highly malignant and only low levels of effective T cells or NK cells are observed in the TME (14, 15). We previously classified ESCC into four different molecular subtypes and only immunogenic tumors showed high levels of cytotoxic cells and other immune cells (33). In this study, we observed that few sequencing reads mapped to TCR regions, consistent with previous observations that the TCR clone size changes dramatically among the infiltrated T cell subtypes and the effective T cells had a low clone size in the ESCC TME (14, 15). Interestingly more than 90% of the aligned reads mapped to BCRs, indicating ESCC showed a unique B cell immunity signature that may be of therapeutic or prognostic value.

Detailed analysis of TIBs showed that the difference between ESCC tumor and adjacent normal samples mainly exists in the IGH chain rather than the light chains. Similar to follicular lymphomas and gastric cancers (22, 41), the V gene usage of TIBs in ESCC is very biased. Almost 37% of rearrangements occurred in the top 5 V genes encoding the IGH chain. As previously reported (43–45), active V genes of IGHV3 subgroups are strongly associated with clinical course and prognosis. We found that IGHV3-74, which is usually rearranged in the normal B-cell repertoire (46), showed a decreased level of rearrangement in ESCC tumor samples, indicating that the low usage of IGHV3-74 can be used to aid ESCC diagnosis. In addition, we observed highly divergent CDR3 sequences among tumor samples, and little similarity of clones existed between tumor and adjacent normal tissues. We further calculated the B cell clonality as 1 – normalized Shannon-Wiener index. Overall, high-frequency CDR3 clones were enriched in tumor samples indicating the TIBs were more diverse with an oligoclonal profile (32). This association may be caused by the accumulation of hyperexpanded B cell clonotypes responding to certain tumor antigens. This kind of B cell clone experiences low level of class switching and rare non-synonymous somatic hypermutation events, and could delay tumor growth. Correspondingly, high clonality of the TIBs is a negative indicator of ESCC survival (multivariable analysis HR: 1.94, **Figure 5B**).

We calculated the clone counts per million sequencing reads (CPMs) for each Ig isotype to adjust for differences in sequencing depth. Consistent with expression quantification determined by RNA-seq data, ESCC tumors showed high levels of IGHG, but low levels of IGHA compared to normal samples. Although subclass switching is very common in ESCC, rare CSR between IgG and IgA subclasses was observed, the majority of CSRs in the ESCC TME happened within IgGs or IgAs. Previously, B cell lineage tracing analysis (47) showed that IgA1 mainly switched from IgM/IgD or IgG1, while IgA2 may be derived from IgG2 or IgG4, in addition to IgA1 and IgM/IgD. We speculate that subclass switching between IgG and IgA was down-regulated in the ESCC TME and IgA may mainly derive from IgM/IgD. In ovarian cancer, higher levels of IgA were observed in TME and associated with improved survival as the TIB-derived IgA redirects myeloid cells against extracellular oncogenic drivers to cause tumor cell death. Moreover, IgA transcytosis could elicit transcriptional changes that antagonize the RAS pathway and sensitize tumor cells to cytolytic killing by T cells (48). The rare IgG/A CSR and low level of IgA in the TME may contribute to the immune suppression within the TME of ESCC.

We examined the prognostic value of tumor-infiltrating B cells and discovered various TIB-associated prognostic biomarkers for ESCC. Strikingly, many of these associations point to IgG2 (**Figure 5B** and **Supplementary Figure S6**). *IGHG2* has a positive prognostic effect in many other cancer types when examined using TCGA data. In melanoma, elevated serum IgG2 was associated with prolonged overall survival and was very predictive for responses to checkpoint inhibitors (49). Therefore, we focused on the prognostic value of B cell types that express IgG2. Both higher *CD138* expression and IGH to *MS4A1* ratio showed a strong association with better survival (**Figure 6A**). Notably, the Ig subclasses analysis confirmed that only the *IGHG2* to *MS4A1* ratio is prognostically associated (multivariable log-rank p: 0.002). Taken together, we speculate the *CD20*-negative IgG2-producing plasma cells constitute a major form of B cell-mediated anti-tumor immunity in ESCC.

Our previous study demonstrated that ESCC could be clustered into four molecular subtypes, which have very different immune environments. The different gene expression profiles and TMEs of each molecular subtype was closely associated with clinical outcomes and the stemness subtype showed the worst 5-year survival. However, there are still patients belonging to other subtypes at high risk of recurrence and death and it would be helpful to distinguish these patients before progression and metastasis.

An integrated analysis of molecular subtyping and TIBs further confirmed that each molecular subtype has unique TIB profiles. The stemness subtype had lowest level of TIBs and other infiltrated immune cells, the immunogenic subtype was enriched in *CD20*-positive non-plasma cells and had the lowest *IGHG2* to *MS4A1* ratio. Most importantly, the *IGHG2*-expressing plasma cell level in the TME positively associated with the prognosis for patients in differentiated, immunogenic and metabolic subtypes, which further distinguished 19 patients at high risk. Thus, combining molecular subtyping and IGHG2 ratio, we maximized the RNA-seq usage to stratify ESCC patients, which could be helpful in identifying clinical interventions to improve clinical outcomes. For those ESCC patients at high risk, developing IgG2 agnostic compounds that focus on the early stage of immune response would be a potentially important new clinical direction. Locking IgG2 into IgG2(B)-like conformations has been shown to promote close packing of *TNFR* (tumor necrosis factor receptor) molecules that initiate signaling and immune activation (50). Additionally, FcγR-independent therapeutics including anti-*TNFR* and anti-*CD40* monoclonal antibodies with human IgG2 agonistic activity have been developed and have showed encouraging clinical outcomes in late stage tumor patients (51, 52).

In summary, the distinct characterization of tumor-infiltrating B cells revealed direct links between Ig class and overall survival in ESCC. The abundance of IgG2-producing tumor-infiltrating plasma cells is a novel biomarker for ESCC prognosis and may provide a novel pathway for the development of new therapeutics to treat ESCC.

## Data Availability Statement

Publicly available datasets were analyzed in this study. This data can be found here: https://ngdc.cncb.ac.cn/search/?dbId=&q=PRJCA001577.

## Ethics Statement

The study protocol was approved by the local ethics committee of the Zhengzhou University and Anyang cancer hospital.

## Author Contributions

YW and YG conceived and directed the project. ZC and SL performed follow up. Bioinformatic analysis and interpretation of the results were performed by ZW with the assistance of JW, ZW wrote the first draft of the manuscript. All authors commented and edited on various versions of the draft manuscript. The authors read and approved the final manuscript.

## Funding

This study was supported by Henan Provincial and Ministry co-constructed youth projects for medical science and technology (SBGJ202103031), the National Key R&D program of China (2016YFE0200800), the Nature Sciences Foundation of China (U1704282 and 81771776). JW and YW also acknowledge support from the Cancer Research UK Centre of Excellence Award to Barts Cancer Centre (C355/A25137].L.C.D is supported by the MRC (MR/V006053/1).

## Conflict of Interest

The authors declare that the research was conducted in the absence of any commercial or financial relationships that could be construed as a potential conflict of interest.

## Publisher’s Note

All claims expressed in this article are solely those of the authors and do not necessarily represent those of their affiliated organizations, or those of the publisher, the editors and the reviewers. Any product that may be evaluated in this article, or claim that may be made by its manufacturer, is not guaranteed or endorsed by the publisher.
