# Supplementary material for: Characterization of the Intra-tumoral B Cell Immunoglobulin Repertoire Is of Prognostic Value for Esophageal Squamous Cell Carcinoma
Source: Front Immunol. 2022 Jun 22;13:896627. doi: 10.3389/fimmu.2022.896627 (PMC9257635; doi:10.3389/fimmu.2022.896627)
Supplement: Supplementary file 1 [file DataSheet_1.pdf]

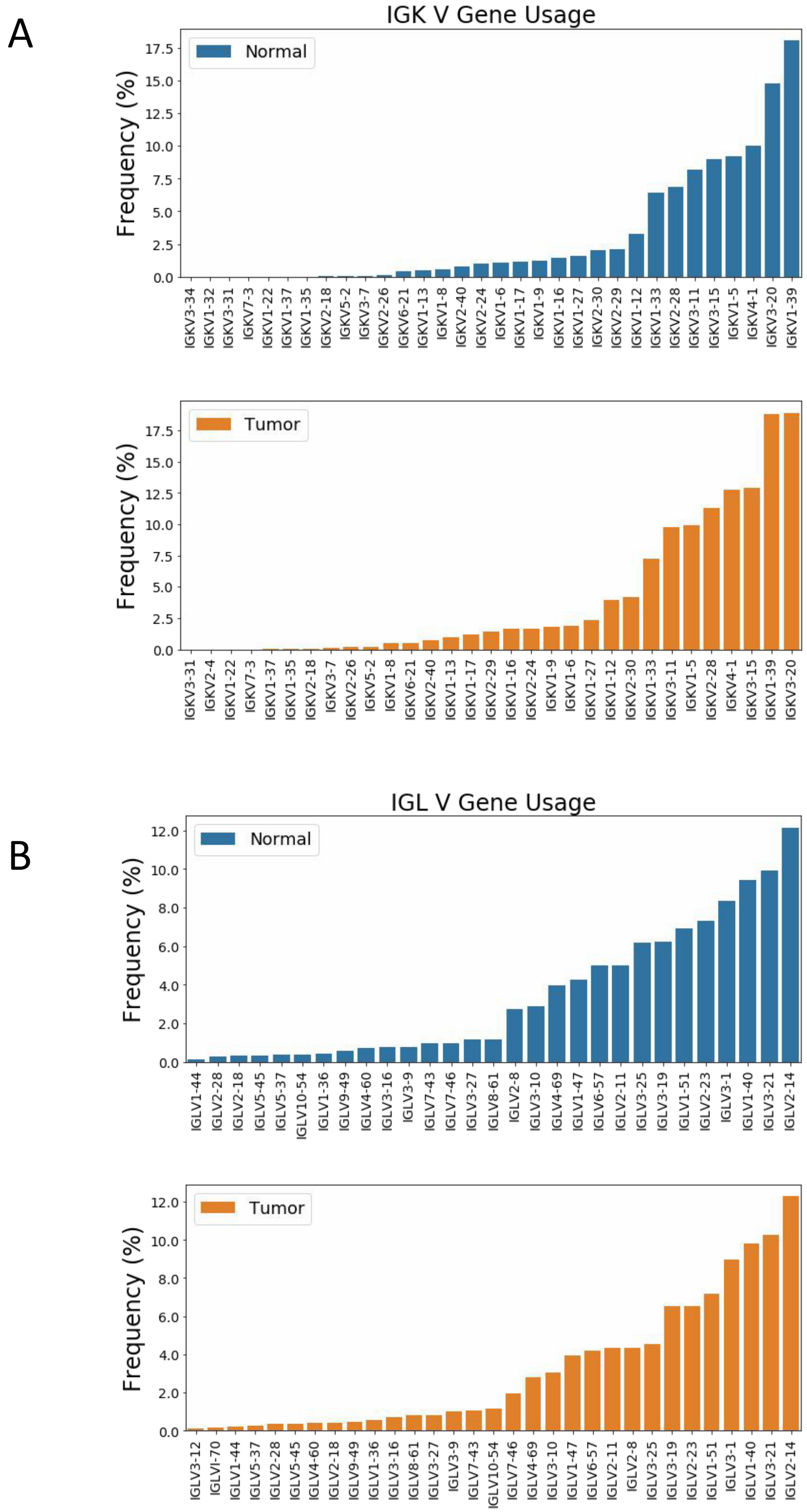

Supplementary Figure S1 V gene usage distribution for IGK chain **(A)** and IGL chain**(B).** Blue bars represent V gene usage in normal samples and Red bars are from tumor samples

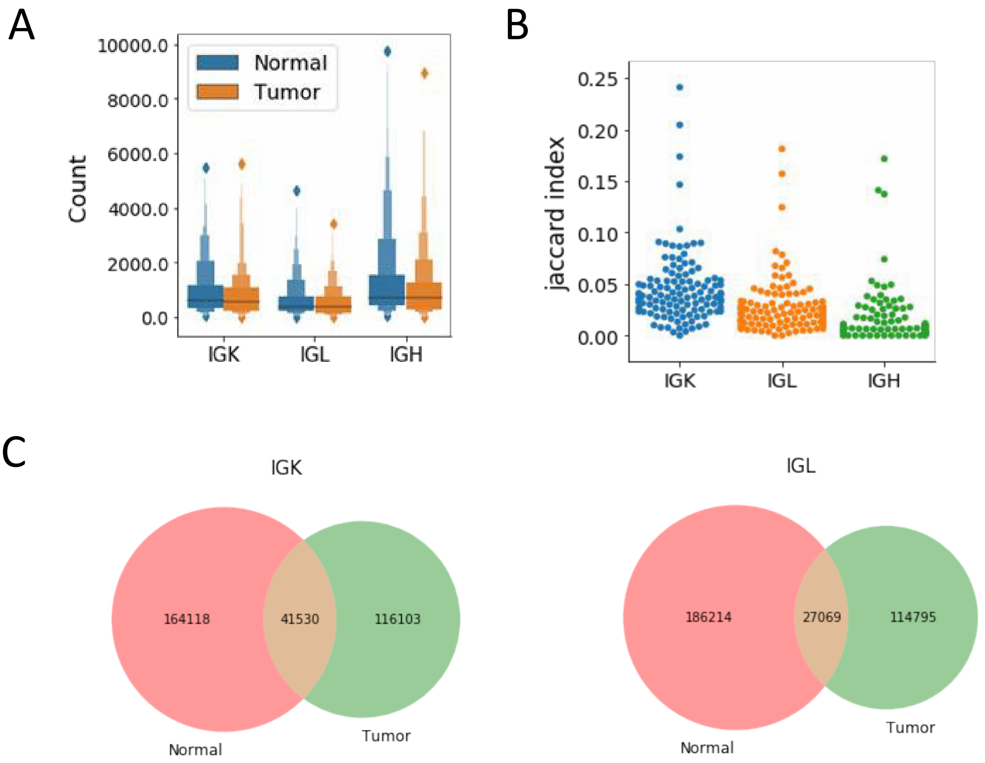

Supplementary Figure S2 CDR3 clone distribution and similarity between tumor and normal samples. **(A)** Boxplot of detected CDR3 clone distribution for IGK, IGL and IGH chains, the width of block represents the relative abundance of clones. **(B)** Jaccard index calculated for each matched tumor and normal samples, the p values was calculated using two-sided Student t test. **(C)** unique CDR3 amino acids determined in tumor and normal samples for the light chains.

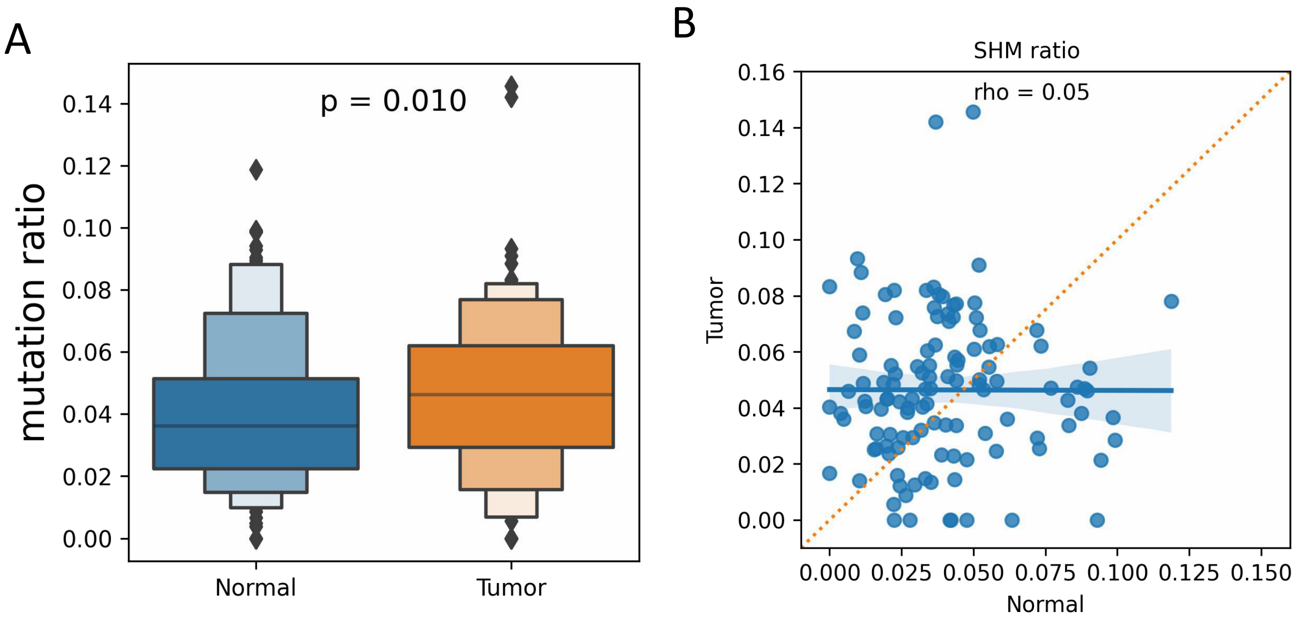

Supplementary Figure S3 Comparison of Somatic Hypermutation (SHM) between tumor and normal samples**. (A)** The mutation ratio distribution of SHMs in tumor and normal samples, Statistical significance was evaluated with Wilcoxon rank-sum test. **(B)** The correlation of SHM ratio between paired tumor and normal samples.

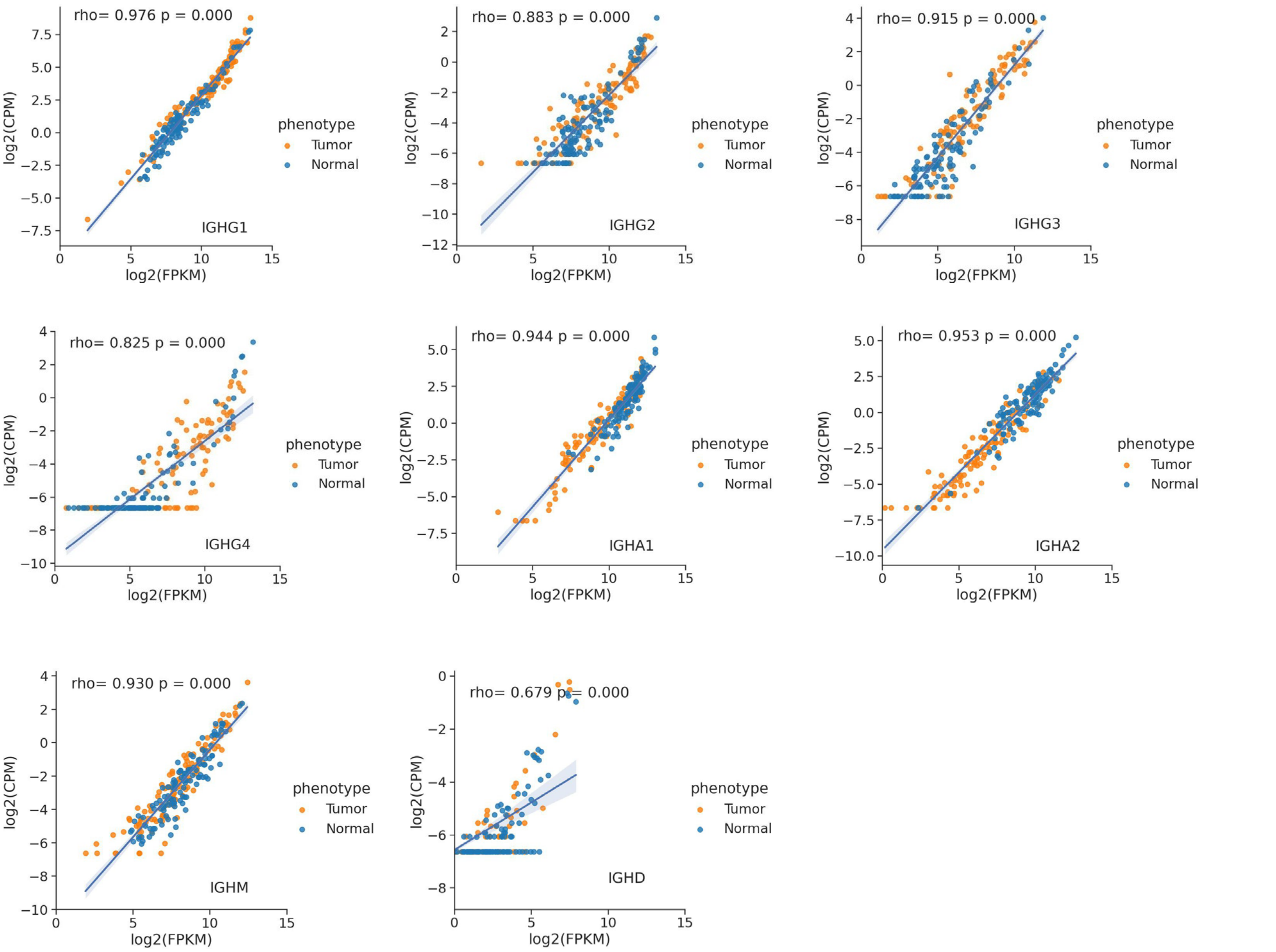

Supplementary Figure S4 The correlation between normalized immunoglobulin abundance represented as CPM and gene expression represented as log2(FPKM).

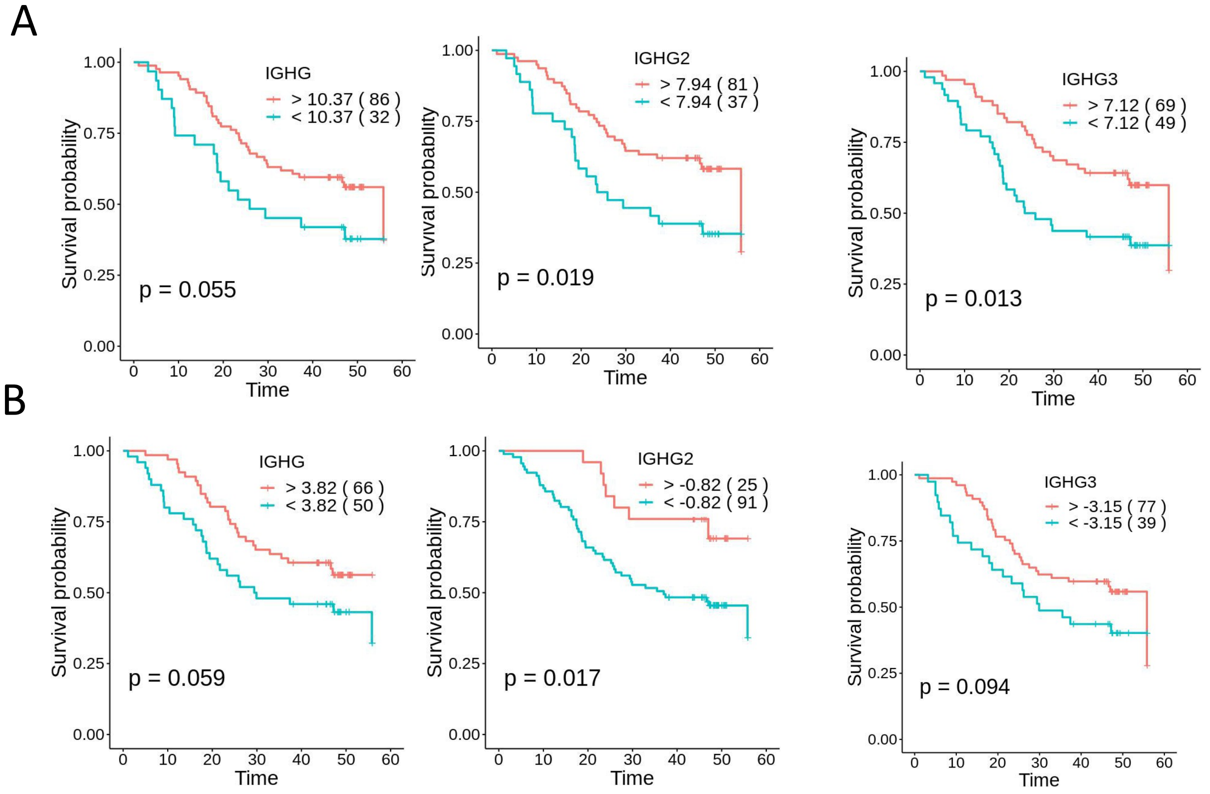

Supplementary Figure S5 Kaplan-Meier overall survival plots for IGHG, IGHG2 and IGHG3 with **(A)** log2(FPKM) and **(B)** log2(CPM). Hazard ratios (HR) were determined with univariable cox proportional-hazards model, and the numbers in brackets consist to the patient number in each group. The cut off for all Kaplan-Meier curve were defined by the surv_cutoff function from the survminer package in the R3.5 environment, the min proportion was set to 0.15.

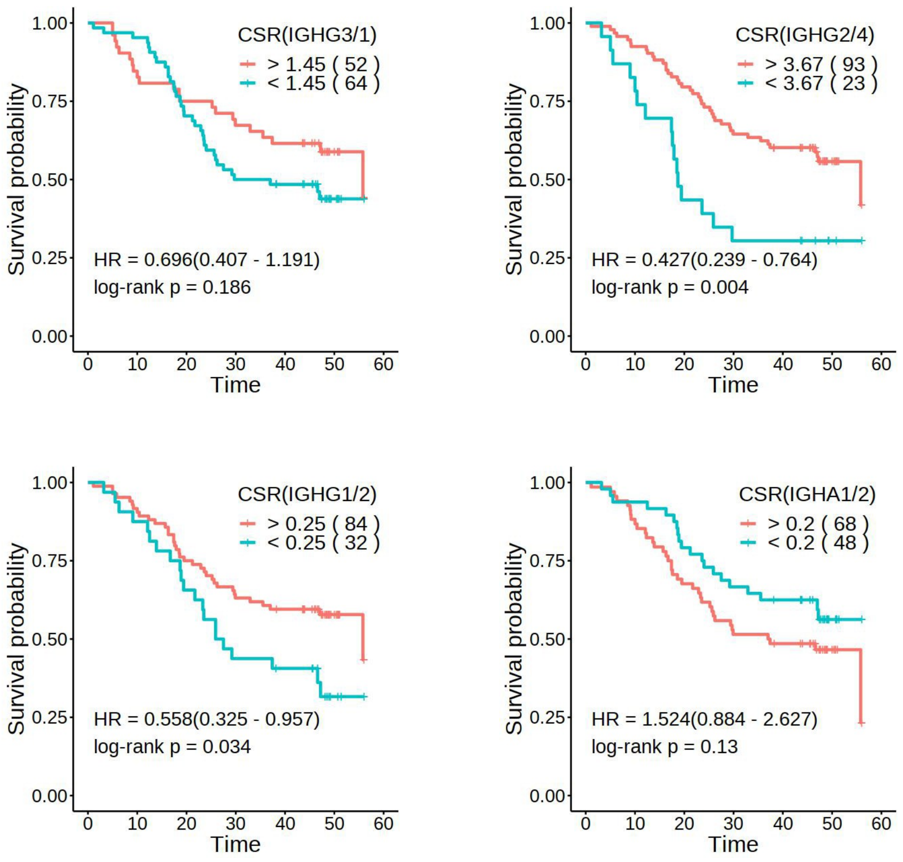

Supplementary Figure S6 Kaplan-Meier overall survival plots for immunoglobulin subclass switch recombination event represented by the ratio of CDR3 clones with reads mapped to two subclass C regions. Hazard ratios (HR) were determined with univariable cox proportional-hazards model, and the numbers in brackets consist to the patient number in each group. The cut off for all Kaplan-Meier curve were defined by the surv_cutoff function from the survminer package in the R3.5 environment, the min proportion was set to 0.15.

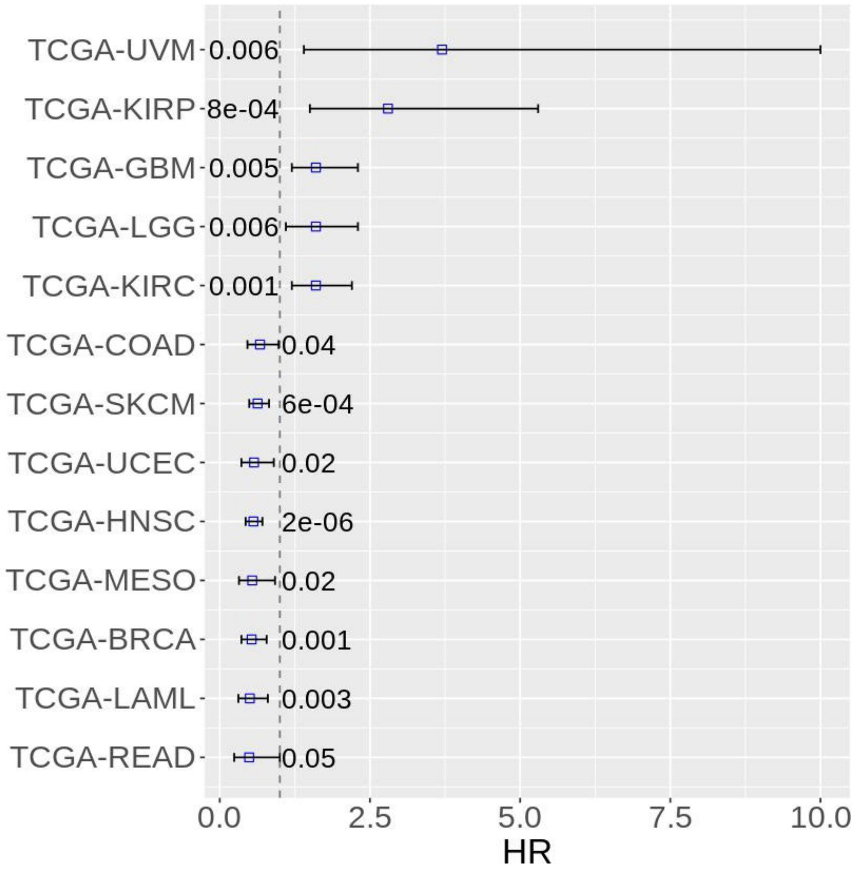

Supplementary Figure S7 The IGHG2 expression and prognosis in TCGA data. Hazard ratios (HR) were determined with univariable cox proportional-hazards model, the numbers marked are the log rank p values. The cut off for all Kaplan-Meier curve were defined by the surv cutoff function from the survminer package in the R3.5 environment, the min proportion was set to 0.15. TCGA：The Cancer Genome Atlas，UVM: Uveal Melanoma, KIRP: Kidney renal papillary cell carcinoma, GBM: Glioblastoma multiforme, LGG: Brain Lower Grade Glioma, KIRC: Kidney renal clear cell carcinoma, COAD: Colon adenocarcinoma, SKCM: Skin Cutaneous Melanoma, UCEC: Uterine Corpus Endometrial Carcinoma, HNSC: Head and Neck squamous cell carcinoma, MESO: Mesothelioma, BRCA: Breast invasive carcinoma, LAML: Acute Myeloid Leukemia, READ: Rectum adenocarcinoma
